# High cholesterol in COVID-19 leading to lung consolidation and bronchiectasis

**DOI:** 10.11604/pamj.2021.39.115.29961

**Published:** 2021-06-09

**Authors:** Tasneem Sajjad Burhani, Waqar Mohsin Naqvi

**Affiliations:** 1 Department of Community Health Physiotherapy, Ravi Nair Physiotherapy College, Wardha, Maharashtra, India

**Keywords:** High cholesterol, COVID-19, lung, bronchiectasis

## Image in medicine

A 60-years-old male with high cholesterol and a Modified Medical Research Council (mMRC) dyspnoea score of 2 was admitted with a history of fever, cough, dyspnoea. Initial chest radiograph (A) showed bronchiectasis in right base with pneumonia in the left base. A diagnosis of COVID-19 was confirmed with positive SARS-CoV-2 RT-PCR on nasal swab. There were some typical findings on CT images. According to the CT images (B), the case was classified as of stage I consolidation with multiple peripheral ground glass opacities with interlobular septal thickening were seen in anterior and inferior lingular segment of left upper lobe, posterior basal segment of left lower lobe, posterior segment of right upper lobe, and medial segment of right middle lobe. Initially he was on IV steroids and therapeutic doses of heparin. He was maintaining his oxygen saturation to 96%. Ten (10) days after hospitalization and normalization of all inflammatory markers, he has Modified Medical Research Council (mMRC) dyspnoea score 1 and oxygen-independent. The patient was asked for follow up in 6 weeks. Due to the mMRC of 1 and short history, it is assumed that the fibrosis was not present prior to the COVID-19 diagnosis. The early use of antifibrotics may prevent this devastating complication, but it is difficult to predict who is likely to progress to pulmonary fibrosis. Early rehabilitation protocol for post-acute COVID-19 patients can minimize dyspnea and shortness of breath even for minimal activities.

**Figure 1:**
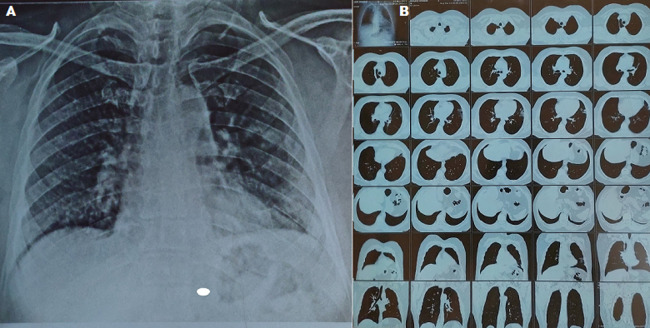
A) honeycomb appearance of bronchiectasis is evident in left upper lobe, posterior basal segment of left lower lobe, posterior segment of right upper lobe, and medial segment of right middle lobe; B) multiple peripheral ground glass opacities with interlobular septal thickening is evident in high-resolution computed tomography (HRCT)

